# Medical Challenge Posed by Retroperitoneal Fibrosis: Case Reports and Literature Review

**DOI:** 10.7759/cureus.6624

**Published:** 2020-01-10

**Authors:** Sergio Cervera-Bonilla, Mauricio Garcia Mora, Paola Rodriguez Ossa, Oscar Messa, Sara Mendoza Díaz

**Affiliations:** 1 Breast and Soft Tissue Surgery, Instituto Nacional de Cancerologia, Bogotá D.C., COL; 2 Oncological Pathology, Instituto Nacional de Cancerologia, Bogotá D.C., COL; 3 Oncology, Instituto Nacional de Cancerologia, Bogotá D.C., COL

**Keywords:** fibrosis retroperitoneal, igg4, idiopathic

## Abstract

Idiopathic retroperitoneal fibrosis (RPF) is a rare fibro-inflammatory disease, with a low incidence worldwide, which occurs around the abdominal aorta and the iliac arteries. It spreads through the retroperitoneum causing ureteral obstruction with associated renal failure and obstruction of other adjacent structures. RPF can be idiopathic or secondary to neoplastic processes, infections, or medications. RPF is considered part of the spectrum of the disease related to immunoglobulin G4 (IgG4) and other autoimmune disorders. Occupational exposure to asbestos and tobacco smoke are important risk factors for the development of idiopathic RPF. The clinical picture is nonspecific, from pain to symptoms due to ureteral compression, this being the main complication associated.

Imaging studies are essential in the diagnosis; computed tomography (CT) and magnetic resonance imaging (MRI) are the most reliable imaging modalities. The goal of treatment is to stop the progression of the fibroinflammatory reaction. The first line of treatment is usually with medical management. Biological agents, such as rituximab and infliximab, have also been used, even with scarce data in the literature. Surgery is usually performed to improve a ureteral obstruction and should always be accompanied by systemic steroid treatment. The conservative approach given by systemic therapy and ureteral stent placement or nephrostomies has been recommended, thus reserving surgical treatment for refractory cases.

We present two clinical cases of idiopathic RPF, one of them associated with IgG4.

## Introduction

Retroperitoneal fibrosis (RPF) comprises a spectrum of rare diseases, characterized by the presence of aberrant fibroinflammatory tissue, which usually develops around the infrarenal portion of the abdominal aorta and in the iliac arteries. It often encompasses neighboring structures such as the ureters and the inferior vena cava. The first description of this disease was made in 1905 by the French urologist Albarrán, who reported the surgical treatment of an extensive retroperitoneal fibrotic process that was causing ureteral obstruction [1]. However, it was not recognized as a clinical entity until 1948, when Ormond made the first publication about this disease in literature [2]. The disease has since been known with a variety of names: fibrous periurethral infection, sclerosing retroperitoneal granuloma, and fibrous retroperitonitis.

Idiopathic RPF has an annual incidence of 0.1 per 100,000 people and a prevalence of 1.4 per 100,000 inhabitants, being more frequent between 40 and 60 years old, with male predominance (3:1) [3]. Idiopathic RPF accounts for two-thirds of cases of RPF, and within this, the disease related to immunoglobulin (Ig) G4; the remaining third is secondary to neoplasms, infections, and medication. The diagnosis is based on imaging studies, such as computed tomography (CT) and magnetic resonance imaging (MRI), always excluding malignant pathology in the first instance.

## Case presentation

Clinical case 1

**Figure 1:**
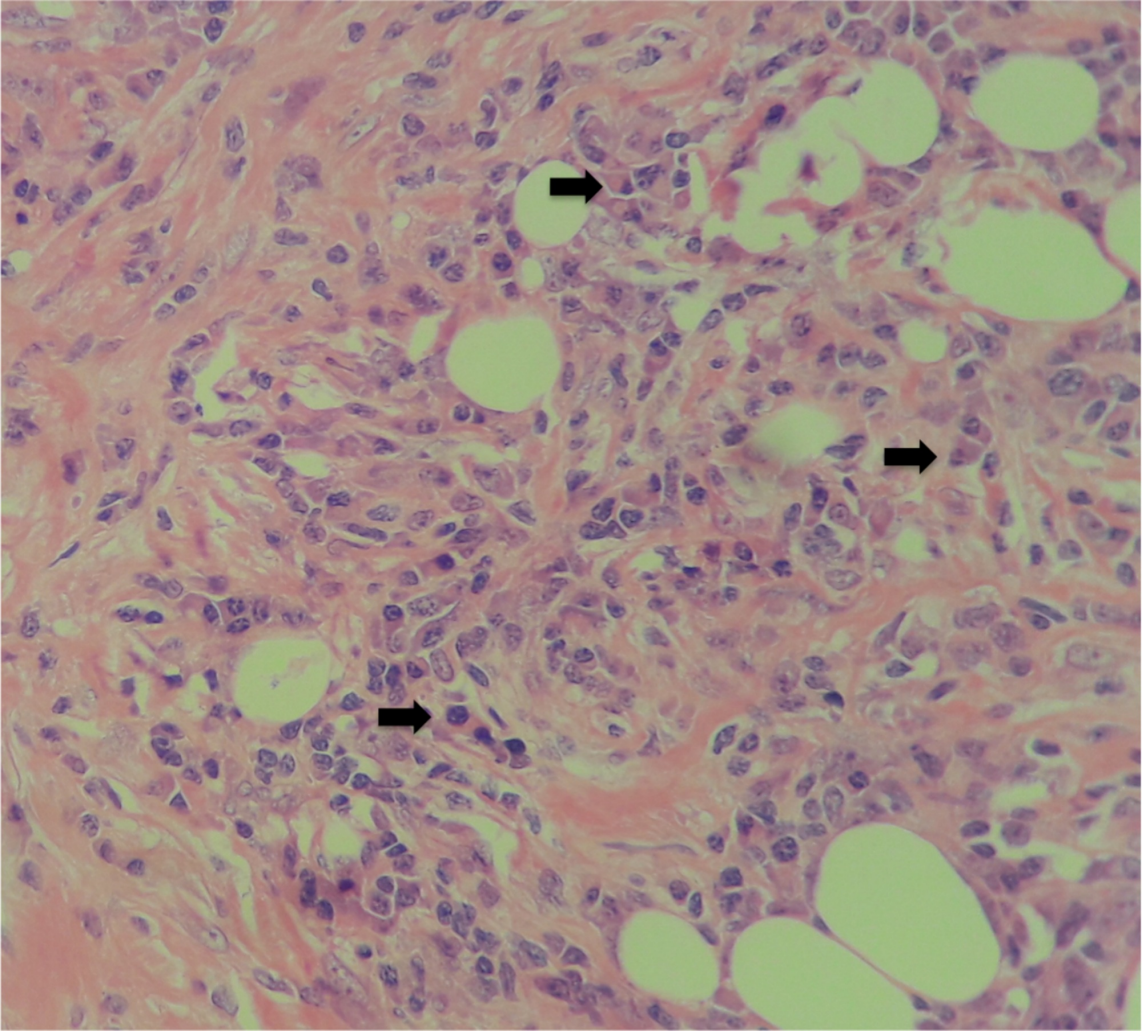
Adipose tissue, fibroblasts, and plasmocytes (arrow). H&E 40X H&E: hematoxylin and eosin

A 54-year-old man presented with a 20-day clinical picture of colic pain in the left flank irradiated to the mesogastrium, which did not improve with outpatient treatment. As background, he revealed repeated urolithiasis, with a report of renal and urinary tract ultrasonography, of a simple renal cyst. CT urogram ruled out lithiasic pathology, showing thickening of the abdominal aorta before bifurcation, and CT reported a simple left renal cyst and retroperitoneal mass in contact with the abdominal aorta, above the bifurcation, with a diameter greater than 4 cm, with no infiltration to the vessel wall and no evidence of distant involvement. The patient underwent laparotomy with findings of a retroperitoneal mass, attached to deep planes, which circumferentially involves the infrarenal abdominal aorta and the intrahepatic vena cava. An incisional biopsy was performed, given the unresectability of the lesion. Surgical pathology reported a hypocellular fusocellular proliferation without atypia, mitosis or necrosis, with trapped fat, with obliteration of vessels with abundant lymphocytes and plasmocytes (Figure 1).

**Figure 2:**
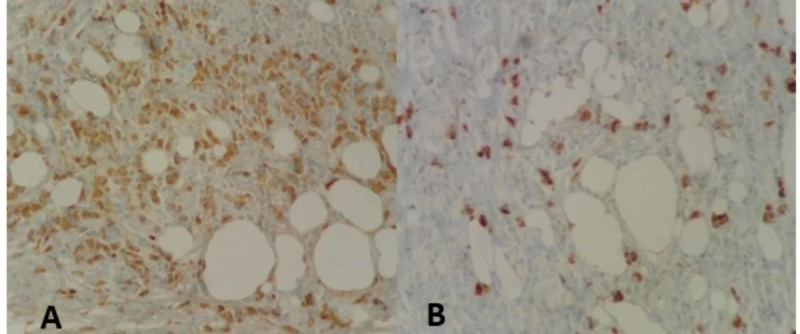
A. Adipose tissue and positive IgG plasmocytes. B. Plasmocytes positive for IgG4 (H&E 40X)

The above was complemented with a genetic study of CDK4 (12q13), MDM2 (12q15) gene amplification by fluorescence in situ hybridization (FISH), for suspected dedifferentiated liposarcoma, which was negative. The requested immunohistochemistry found negativity for ALK, Desmin, P.16, S100, CD34, AE1/AE3, HMB45, CD117, CD31, and CAM5.2, and frequent IgG4 plasmocytes (Figure 2), with areas of more than 50 IgG4 plasmocytes per CAP, although the IgG4 / IgG ratio is not greater than 40%. Additional IgG and IgG4 markers were performed, which were positive.

Pathology findings showed a lesion of mesenchymal appearance, composed of a hypocellular proliferation of reactive fibroblasts, accompanied by inflammatory lymphoplasmacytic infiltrate. These findings raised several differential diagnoses that will be discussed below: well-differentiated liposarcoma is a neoplasm that occurs in this location and in that age group, however, the exclusive perivascular arrangement is not frequent and even less around the aorta. From the histological point of view, we did not find lipoblasts or dispersed atypical stromal cells. In addition, p16 by immunohistochemistry and FISH was performed for MDM2 and CDK4, which were negative. Another option that was raised was the inflammatory pseudotumor; however, this pathology is more frequent in adolescents and young adults. The immunohistochemical study for ALK was negative and, in this pathology, the accompanying inflammatory infiltrate tends to be mixed with neutrophils and eosinophils, and no elevation of IgG4 would be found. Other remote possibilities were: lymphoplasmacytic lymphoma, sarcomatoid metastatic carcinoma, leiomyosarcoma, desmoplastic melanoma, and gastrointestinal stromal tumor (GIST). But complementary immunohistochemical studies for CAM 5.2, CD34, CD117, Desmin, S100, and HMB45 were negative. With the previous findings, a definitive diagnosis was made of a retroperitoneal fibro-plasmacytic lesion compatible with IgG4 disease. The patient continued postoperatively, with good clinical evolution, so he was treated in an ambulatory manner with endocrinology follow-up.

Clinical case 2

**Figure 3:**
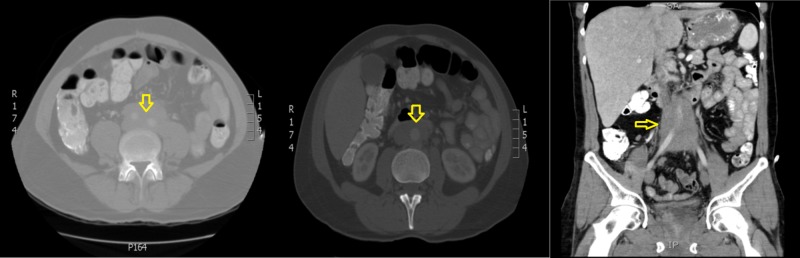
Computed tomography of the abdomen Retroperitoneal mass of infrarenal location, compromising the para-aortic, interaortocaval space in front of the aorta, extending to the aortoiliac bifurcation, with well-defined contours, surrounding vascular structures without infiltrating them, 99 x 38 x 55 mm, and associated with left hydronephrosis

A 48-year-old male patient presented with no relevant history and a clinical picture of two months' evolution, characterized by a predominantly left abdominal pain, with the sensation of a mass. CT of the abdomen showed a retroperitoneal mass of infrarenal location, compromising the para-aortic, interaortocaval space in front of the aorta, extending to the aortoiliac bifurcation, with well-defined contours, surrounding the vascular structures without infiltrating them, 99 x 38 x 55 mm, and associated with left hydronephrosis (Figure 3). No distant involvement was evident.

A Tru-Cut biopsy was performed, with a histopathological report of “fibro-fatty tissue with changes due to fat necrosis, poor reactive lymphoid tissue, negative for epithelial malignancy,”; additional flow cytometry was performed, which was negative for lymphoid neoplasia, T lymphocytes with adequate CD4-CD8 reaction, polytypic B lymphocytes without aberrant expression of CD5, and low plasma cell population within normal parameters. Tumor markers, serology, immunoglobulin levels, and bone marrow studies were negative. Suspecting IgG4-mediated disease, an analysis of serum IgG4 levels was performed, which was negative. Given the conclusive biopsy, an excisional biopsy was performed by laparotomy, with a histopathological report of “lesion of fibroinflammatory appearance, with dense fibrosis, with some larger fibroblasts surrounded by small lymphocytes of interstitial location and forming occasional lymphoid aggregates, with some secondary follicles with a germinal center; additionally, scattered plasmocytes and eosinophils are recognized. There is no evidence of atypia, tumor necrosis, microorganisms, multinucleated giant cells, granulomas, or morphological criteria of epithelial malignancy or lymphoproliferative neoplasia.”

Immunohistochemistry studies show small, predominantly T-positive lymphocytes with CD3. Occasional secondary lymphoid follicles show positive germ centers with CD20, CD10, and BCL6, and negatives with BCL2, with high and polarized Ki67. CD30 shows few immunoblasts. Plasmocytes are positive with CD138 and polytypic with Kappa and Lambda light chains. The IgG and IgG4 ratio is not altered (<0.4). Cyclin D1 is negative, AE1/AE2 cytokeratins do not show epithelial cells, and smooth muscle actin is positive in myofibroblasts. Additional studies with desmin, beta-catenin, and ALK are negative. Ki67 in the fibrous component is less than 5%. It is concluded that due to morphological findings and immunohistochemistry, correlating with imaging and intraoperative data, they lead to mass-forming fibroinflammatory disorders of the idiopathic RPF type. Medical treatment was initiated with corticosteroids at a dose of 1 mg/day and clinical follow-up without associated progression or morbidity episodes.

## Discussion

The term retroperitoneal fibrosis (RPF) commonly refers to a clinical-pathological entity characterized by a mainly sclerotic tissue that develops in the periaortic and peri-iliac retroperitoneum and often encloses structures such as the ureters and the inferior vena cava [3]. RPF covers the idiopathic form (> 75% of cases) and secondary forms to malignancies, infections, medication, radiotherapy, or other conditions [4]. Idiopathic RPF is a very unusual entity, with an estimated incidence of 0.1 - 1.3 cases / 100,000 people per year and a prevalence of 1.4 cases / 100,000 inhabitants [4]. There are no standardized criteria for classifying idiopathic RPF, but it is currently included in the spectrum of chronic periaortitis (PC) together with perianeurysmal RPF and inflammatory abdominal aortic aneurysms since all these forms have similar histological features and clinical presentation. Idiopathic RPF (or PC) can be isolated or develop in the context of a systemic immune-mediated disease [5].

Idiopathic RPF is part of the spectrum of the disease related to immunoglobulin G4 (IgG4), an immune-mediated disorder that can affect different organs (pancreas, gallbladder, salivary glands) and that is histologically marked by a lymphoplasmacytic infiltrate rich in plasma cells carrying IgG4, fibrosis, tissue eosinophilia, and obliterating phlebitis and is also characterized by elevated serum IgG4 levels in a significant proportion of cases; some cases of idiopathic RPF have such histological and clinical characteristics and, therefore, can be classified as related to IgG4 [6], although the exact proportion of IgG4-related cases of the total number of idiopathic RPF cases is still unclear. As a multifactorial entity, it can be associated with rheumatoid arthritis, ankylosing spondylitis, antineutrophil cytoplasmic antibodies (ANCA)-associated vasculitis, systemic erythematosus lupus, psoriasis, and membranous nephropathy (MN). In our case, although the findings in pathology are not specific for IgG4-associated disease, the presence of a benign fibroblastic proliferation mixed with abundant plasmocytes makes it suspicious; with immunohistochemistry, we could rule out myeloma (with the expression of CD56) and demonstrate the predominance of IgG plasmocytes with frequent IgG4. Although in this case, the IgG/IgG4 ratio was not as expected, the union of the imaging triad, elevated serum IgG4, and histology allowed the proposed diagnosis.

Different mechanisms have been proposed to explain the pathogenesis of idiopathic RPF. Starting as a manifestation of a systemic autoimmune disease and that which may arise as a primary aortitis that subsequently causes a periaortic fibroinflammatory response. Clinical findings, such as the presence of systemic symptoms, association with other autoimmune disorders, as well as the good response to immunosuppressive therapies, conform to this systemic theory mediated by the immune system [7]. Multiple factors may contribute to this process. Martorana et al. demonstrated a strong association between idiopathic RPF and HLA-DRB1 * 03, an allele related to many autoimmune conditions such as type 1 diabetes, myasthenia gravis, and autoimmune thyroiditis [8]. Occupational exposure to asbestos and tobacco smoke are two important risk factors for the development of idiopathic RPF [9]. With respect to drugs, ergotamine increases endogenous serotonin levels and has been suggested to lead to fibrotic reactions through the proliferation of myofibroblasts [10]. Malignant tumors are often listed as possible causes of secondary RPF. In most of these cases, RPF is the consequence of a lush desmoplastic response of retroperitoneal metastases (prostate, breast, or colon carcinoma) or of a primary retroperitoneal tumor (Hodgkin and non-Hodgkin lymphomas, inflammatory myofibroblastic tumor, a sclerosing variant of well-differentiated liposarcoma, and various types of sarcomas) [11]. In the case of infection-related RPF, the disease is usually secondary to the local spread of a contiguous infectious site (spinal or paraspinal abscesses in patients with tuberculosis) or to an immune response triggered by a remote infection. In addition to Mycobacterium tuberculosis, which has been reported as an etiologic agent, actinomycosis or histoplasmosis can sometimes represent primary infections [12].

In our case, they did not present associations with environmental, infectious, or medicinal factors. Histologically, fibrous and inflammatory tissue predominates in idiopathic RPF, which is composed of an extracellular matrix with type I collagen fibers. IgG4-related RPF is characterized by plasma cell proportion > 40% in addition to eosinophil infiltration [13].

The correct diagnosis is made only when obstructive uropathy or renal failure occurs (42%-95%). In most patients, the presenting symptom is dorsal, flank, and/or abdomen pain. Other rare symptoms include fever, testicular pain, abdominal angina, intermittent claudication, edema, or macroscopic hematuria. Urinary tract obstruction can lead to acute hypertension in some patients, possibly due to an increase in renin release [14]. In our case, ureteral involvement associated with unilateral hydronephrosis was observed. 

Inflammatory markers, such as erythrocyte sedimentation rate (ESR) and C-reactive protein (CRP), are elevated in more than half of the patients and do not help differentiate idiopathic or secondary RPF [15]. Unlike patients with idiopathic RPF, those with IgG4 generally have a history of chronic allergic conditions (atopia, eczema, asthma, and eosinophilia in peripheral blood) and tend to develop tumor-like lesions, which may be responsible for tumor inflammations of the organs affected. One of the most important characteristics of IgG4 is the presence of high serum IgG4 levels. There are few data on serum IgG4 levels in patients with extrapancreatic IgG4. Most patients have elevated serum IgG4 levels but the range varies. The published data correlates with the findings of our case, where it is reported that approximately 30% of patients have normal serum IgG4 levels despite the presence of classic histopathological and immunohistochemical findings, which include a significant infiltration of plasma cells with IgG4 [5]. There are no laboratory tests that can identify the secondary form of RPF; however, for example, high concentrations of neoplastic markers are identified in cases secondary to certain malignant tumors [16].

Imaging studies are essential in the diagnosis and treatment of RPG and can help differentiate between idiopathic and secondary disease. Ultrasound should be done as the first line of study, especially when evaluating a patient with renal failure. On ultrasound, idiopathic RPF presents as a hypoechoic or isoechoic mass, which can involve the ureters and, therefore, cause unilateral or bilateral hydronephrosis. CT and MRI are the most reliable imaging modalities for the diagnosis of idiopathic RPF [16].

In CT without contrast, it usually appears as a homogeneous, isodense plate with muscle, which surrounds the lower abdominal aorta and the iliac arteries, wrapping the ureters and the inferior vena cava. In contrast, RPF secondary to malignant diseases tends to displace the aorta anteriorly and the ureters laterally. Magnetic resonance provides a better definition. Idiopathic RPF is hypointense in T1-weighted images; in weighted T2, its intensity is high in the early or active stages of the disease due to tissue edema and hypercellularity and low in the last stages [16]. Positron emission tomography with fluorodeoxyglucose is not useful for the diagnosis of RPF due to its low specificity but can be taken into consideration for assessing disease activity [17]. When imaging studies do not show the typical findings of idiopathic RPF, a tissue biopsy is often required; biopsy may also be recommended in patients refractory to conventional steroid therapy. Multiple biopsy techniques have been used in RPF sampling, including open or laparoscopic retroperitoneal biopsy. However, in malignant RPF, metastatic cells generally disperse so diffusely in fibrotic tissue that multiple deep surgical biopsies are needed [18].

The goal of idiopathic RPF treatment is to stop the progression of the fibroinflammatory reaction in order to inhibit or improve the obstruction of the ureters or other retroperitoneal structures, the acute phase reaction, and its systemic manifestations. Corticosteroids are the first-line therapy, suppressing the synthesis of most cytokines involved in the acute phase reaction, reducing the inflammation and inhibition of collagen synthesis and maturation [19]. Corticosteroids achieve an improvement in symptoms and often lead to a reduction in the size of the retroperitoneal mass and a resolution of obstructive complications. Remission rates after steroid therapy range from 75% to 95%; the average reduction in mass thickness is around 50%. Glucocorticoids are rapidly effective and most of the radiographic response is observed within the first weeks of treatment. After a month of therapy, a reassessment of disease activity is recommended along with a morphological evaluation of the mass. If remission is obtained, prednisone can be progressively reduced to 5-10 mg daily within three to four months and then maintained for an additional six to nine months. 

When there is a contraindication for glucocorticoid therapy, a good alternative may be the use of tamoxifen [5]. Immunosuppressants are also often used. Among these, cyclophosphamide and azathioprine can induce stable disease remissions and mass regression, although they can be very toxic. Idiopathic RPF is, in fact, a chronic relapse disorder, with relapse rates of up to 72% [13]. Tamoxifen has been used but its effectiveness is uncertain. In the only randomized controlled trial conducted in patients with idiopathic RPF, 36 out of 39 patients who obtained remission after induction therapy with Prednisone were randomly assigned to a gradual decrease in prednisone or switch to tamoxifen (0.5 mg/kg/day) for eight months. Relapses were more frequent in the tamoxifen group, and the difference between the groups in relapse rates was significant both at the end of treatment (month 8) and at 26 months of follow-up, which is why therapy is recommended second-line as well as immunosuppressants, in steroid-refractory patients [16]. Biological agents have been used in the case of refractory idiopathic RPF. The agents used were rituximab, infliximab, and tocilizumab, but data is still scarce. Idiopathic RPF in the context of IgG4 tends to respond rapidly after six to nine months of glucocorticoid therapy, but rituximab has been used effectively in refractory cases [5].

Surgery is usually performed to improve ureteral obstruction; Open ureterolysis with intraperitoneal transposition and omental envelope of the ureters is considered the best surgical approach [20]. Surgery allows multiple retroperitoneal biopsies, but surgical treatment addresses only ureteral obstruction; it does not prevent disease progression or recurrence. Surgery must be accompanied by systemic steroid treatment. The conservative approach consisting of systemic treatment associated with ureteral stent placement or nephrostomies has been recommended, thus reserving surgical treatment for refractory cases [16]. After the onset of systemic therapy, the follow-up is clinical, and additionally, ultrasound is useful for the control of ureteral obstruction, but CT and MRI are for the evaluation of changes in retroperitoneal tissue size. It is estimated that ureteral obstruction is recurrent in half of the patients who undergo surgery and in 10% of those who were treated with surgery and steroids. The treatment of secondary forms of RPF is the treatment of the cause such as drugs, of which suspension resolves the disease; however, steroids can be used as an associated treatment.

## Conclusions

Retroperitoneal fibrosis is a rare disease that is difficult to diagnose and constitutes a therapeutic challenge for retroperitoneal diseases. The diagnosis is histopathological, where an inflammatory infiltrate is initially shown by lymphocytes, plasmatic cells, and macrophages. The possibility of a disease related to IgG4 should always be ruled out and its serological ratio established for confirmation. The diagnostic images of choice are CT and MRI, which show the classic compromise surrounding the aorta and ureter. The first-line management is medical with glucocorticoids; when there is a contraindication, tamoxifen, immunosuppressants, and biological agents are proposed. Surgical management has been reserved for refractory cases; conservative treatment with systemic therapy being the standard treatment. RPF is a benign entity with biological aggressiveness and low incidence but high morbidity, so it always needs to be managed in a center of high complexity, with experience in retroperitoneal tumors.
